# Experiences and Impacts of Intimate Partner Violence Against Men in Northern Ireland: Qualitative Findings from the Male Experiences of Intimate Partner Violence Study

**DOI:** 10.3390/bs16061007

**Published:** 2026-06-16

**Authors:** Eric Spikol, Emily McGlinchey, Cherie Armour

**Affiliations:** 1 School of Psychology, Ulster University, Cromore Road, Coleraine BT52 1SA, Northern Ireland, UK; e.spikol@ulster.ac.uk (E.S.); e.mcglinchey@ulster.ac.uk (E.M.); 2 Stress Trauma and Related Conditions (STARC) Research Centre, School of Psychology, Queen’s University Belfast, David Keir Building, 18-30 Malone Road, Belfast BT9 5BN, Northern Ireland, UK

**Keywords:** intimate partner violence, IPV, mental health, victimisation, Northern Ireland, male mental health, public health

## Abstract

Intimate partner violence (IPV) affects individuals of all genders and can result in adverse physical, psychological, and social outcomes. Experiences of IPV in men remain understudied when compared with those of cisgender women, leading to considerable gaps in understanding of prevalence, experiences, disclosure, and outcomes. The Male Experiences of Intimate Partner Violence Study (ME-IPV Study) was designed to explore: nature of IPV experiences, physical and psychological impacts, barriers to reporting/disclosing, experiences of disclosure, experiences of support, and support needs in a Northern Ireland (NI) context. This mixed-method study utilised data from N = 10 qualitative interview participants (quantitative results reported separately), analysed using an Interpretative Phenomenological Analysis (IPA) framework. Participants described experiencing multiple forms of IPV, with coercive control, psychological and institutional abuse being highly prevalent. Detrimental effects of their experiences included diagnoses of anxiety, depression, and PTSD, physical symptomology, the advent/exacerbation of multiple health conditions, and suicidal ideation. Barriers to care were primarily a lack of dedicated care pathway, concerns over being believed, and stigmatic barriers. Experiences of disclosure were mixed: positive with family/friends and negative with police and institutions of state. Male experiences of IPV in NI are a significant public health issue and it is evident that the impacts of IPV on men’s physical/mental health and wellbeing are profound.

## 1. Introduction

### 1.1. Background

Intimate partner violence (IPV) can be defined as any act of “physical violence, sexual violence, stalking and psychological aggression (including coercive tactics) by a current or former intimate partner” (Breiding et al., 2015), and has been identified as a significant health concern worldwide. IPV is considered to be an adverse or traumatic life event (Wathen & Mantler, 2022). It affects individuals of all genders and can result in adverse physical, psychological, and social outcomes (McNeil et al., 2023). To date, IPV against cisgender women and its impacts have been widely researched, but the experiences of men remain understudied (Scott-Storey et al., 2023; Taylor et al., 2022). There is no empirical consensus regarding IPV experience prevalence among men, variation in the type of IPV experiences, nor in any differences or unique needs resulting from these experiences (Scott-Storey et al., 2023; Taylor et al., 2022). This dearth of research leaves a significant gap in our knowledge and understanding of IPV among men.

The global prevalence of IPV in men is estimated to range from approximately 17% (Gubi & Wandera, 2022) to 20% (Lanre et al., 2014). A recent review of prevalence and impacts of IPV in men/boys (McGlinchey et al., 2023) found that results varied widely based on location, study methodology, definition of IPV, and a range of other factors. Under-reporting also affects these rates, as many men who have experienced IPV are unaware their experiences meet the definition of abuse (Bates, 2020a, 2020b; Taylor et al., 2022). They may fail to report due to self and societal stigma, retaliation concerns, and fear of not being believed (Walker et al., 2020; Bates, 2020a; Taylor et al., 2022).

In the UK, prevalence rates of domestic violence (DV; abuse perpetrated by any family, including but not specific to intimate partners), including IPV, were estimated at 13.8% in England and Wales (Office for National Statistics, 2020), concordant with the UK-wide findings of 13.8% of men having experienced DV (The Mankind Initiative, 2021). As this references the wider umbrella definition of DV, it is likely these approximations underrepresent the true rates of IPV in UK men. Exploring this in NI is difficult. While the Police Service of Northern Ireland (PSNI) publishes yearly statistics describing DV in NI, they do not differentiate violence between family members from intimate partner violence. Thus, the PSNI have reported that 32% of all DV victims in 2021 were male (Police Service of Northern Ireland, 2022a), and as these statistics only cover reported crimes and as men are significantly less likely to report (Dutton & White, 2013; Walker et al., 2020), the true prevalence of IPV in NI men remains unknown.

It is pertinent to recognise that men are not a homogeneous group regarding their risk and experiences of IPV. Indeed, data from England and Wales (Office for National Statistics, 2020, 2022) has shown LGBTQ+ men were twice as likely to experience IPV compared to heterosexual men (Ministry of Justice, 2022). While the popular perception of male IPV victimisation focuses on a heteronormative couple with a female perpetrator (Baker et al., 2013; Cannon, 2015), a recent meta-analysis of 52 studies, covering approximately 32,000 participants, estimated a pooled prevalence rate of 33% regarding IPV victimisation in male/male relationships (Liu et al., 2021). Likewise, an added barrier to reporting of IPV experiences is the always present threat of ‘outing’ by the perpetrator, i.e., disclosure of one’s LGBTQ+ status (Callan et al., 2021), especially in places where being LGBTQ+ is illegal and/or highly stigmatised (Lanre et al., 2014; Hall et al., 2020; Okanlawon, 2018; Ogunbajo et al., 2022).

### 1.2. Impacts of IPV

The impact of IPV on an individual’s physical and mental health can be profound. In addition to physical injuries, the relationship between trauma, especially cumulative trauma, such as a sustained pattern of IPV, and adverse health outcomes, is well-established (Coker et al., 2002; Lagdon et al., 2014; Hines & Douglas, 2015; Brooks et al., 2020). While there is an established association between prolonged IPV experiences and adverse psychological, cognitive, and neurological outcomes in women due to traumatic brain injury (E. Valera & Kucyi, 2017, E. M. Valera et al., 2019; E. M. Valera, 2018; Daugherty et al., 2021, 2024), this association has not been explored in men. IPV victimisation in men has been associated with anxiety and depression (Próspero, 2007; Scott-Storey et al., 2023; Macassa et al., 2023), suicidal ideation (Randle & Graham, 2011), post-traumatic stress disorder (PTSD) (Hines, 2007; Lagdon et al., 2014, 2023; MacManus et al., 2022), and alcohol/substance misuse (Coker et al., 2002). Participants in a qualitative study on the life impacts of IPV in men (Sita & Dear, 2021) described decreased self-worth, self-efficacy, and concentration, and increased social isolation.

IPV impact also varies by type of abuse. Sexual violence has been found to be highly associated with depression, PTSD, and poor physical health (Hines & Douglas, 2016a). Physical violence was associated with chronic illness/mental illness, with worse physical health outcomes occurring when psychological violence was also present (Coker et al., 2002). Psychological violence has been associated with PTSD, depression, suicidal ideation, poor physical health, and decreased wellbeing (Shorey et al., 2012; Hines & Douglas, 2016b; Machado et al., 2020; Scott-Storey et al., 2023). A study of Italian men reported that although participants described quite severe physical violence as detrimental to their wellbeing, many reported that the psychological violence had a more detrimental impact (Entilli & Cipolletta, 2017).

In addition to PTSD, IPV victimisation has also been associated with an increased CPTSD risk in both women (Fernández-Fillol et al., 2021) and men (Gilbar et al., 2019). As each instance of IPV can be considered a separate adverse life event, prolonged experiences increase PTSD/CPTSD risk in a cumulative dose-response relationship (Wilker et al., 2015; Contractor et al., 2018).

#### Disclosure, Barriers to Help-Seeking, and Support in Northern Ireland

Men are more likely to either not report due to psychosocial and cultural influences, or to not recognise their experiences as IPV (Bates, 2020b; Taylor et al., 2022). Thus, lack of disclosure masks the actual scope of the problem. Correspondingly, institutional financial stakeholders are unwilling to commit funds to organisations or statutory initiatives for what they may perceive to be a ‘minor issue’. Research relying on official disclosure/prevalence rates can be problematic due to under-reporting and studies are often limited to samples taken from clinical or help-seeking populations (Ali et al., 2021; McGlinchey et al., 2023).

Lack of disclosure in men can be driven by the fear that their experiences would be minimised/ignored/not believed, or they themselves would be accused of perpetrating abuse (Walker et al., 2020; Bates, 2020a; Taylor et al., 2022). Bates (2020a) described participants’ experiences of being mocked by police and health professionals, and being told to “man up” or they “must have deserved it” by support staff. Men may be met with suspicion when they disclose (Dutton & White, 2013) and forced into a network of institutions predisposed to stereotype men as aggressive perpetrators and women as fragile victims incapable of perpetration (Barber, 2008). Reluctance can also be affected by the family situation, as legal systems in many countries favour women in custody disputes (Brown, 2004; Tsui, 2014), with many men remaining in abusive relationships out of fear they will be kept from their children (Berger et al., 2016; Bates, 2020a; Moore, 2021). Increased cumulative trauma has also been associated with hesitance in help-seeking and increased perception of barriers to care (Spikol et al., 2024a).

Gender stereotypes have influenced how IPV has been conceptualised within many societies (Bates & Carthy, 2020). The pervasive notion that being male means conforming to a set of behaviours espousing ‘strength’, ‘stoicism’, and ‘self-reliance’ is present in many cultures, with any deviation seen as being ‘weak’, ‘unmanly’, or ‘feminine’ (Tsui, 2012). A survey amongst support agencies in the United States (Tsui et al., 2010) described several barriers influencing low rates of disclosure and help-seeking among men: (i) perception of services as female oriented, (ii) shame and embarrassment, (iii) denial over the abuse, (iv) stigmatic beliefs, and (v) fear. As help-seeking is associated with better post-IPV outcomes (Douglas & Hines, 2011) these barriers constitute a significant public health issue.

Even when NI men make the decision to disclose and/or seek support, they face a lack of dedicated support services. At present, there is no IPV refuge for men in NI, meaning that men leaving an unsafe home environment risk homelessness. Several IPV/DV charities operate in NI to provide what support they can, including Men’s Alliance, Men’s Action Network, and the Men’s Advisory Project, but these have suffered in terms of funding due to years without a sitting Executive and recent cuts to third sector budgets. While some stigmatic beliefs around help-seeking have declined in NI (O’Neill & O’Connor, 2020), potentially due to awareness campaigns, stigma remains an enduring barrier to support (Betts & Thompson, 2017; Spikol et al., 2024a).

These challenges have a direct impact on service provision, governmental strategies/policies, and implementation of support initiatives. On a national level, the UK has adopted a framework to guide policy on IPV focusing on women/girls (Crown Prosecution Service, 2016), which mentions male victims of IPV once and links to a separate government statement on male DV/sexual assault victimisation unspecific to IPV. Locally, the NI Executive has proposed two strategies, ‘Tackling Violence Against Woman and Girls Action Plan’, which only applies to females and the ‘Domestic and Sexual Violence and Abuse Strategy’, which covers both males and females (Police Service of Northern Ireland, 2022b). Both documents have completed public consultation and are now pending response from the Executive.

### 1.3. Study Aims

It is evident that the prevalence rates, experiences, and impacts of IPV among men in NI “remain understudied and poorly understood” (McGlinchey et al., 2023) and a campaign of targeted local research is urgently needed to form a better understanding of the impacts of IPV, which can be drastic and potentially long-term (Coker et al., 2002; Walker et al., 2020; Taylor et al., 2022). The Male Experiences of Intimate Partner Violence Study (ME-IPV Study) was designed as a mixed methods study to address these issues. It utilised an online quantitative survey to explore prevalence, types of experiences, barriers to help-seeking, attitudes towards disclosure, and health/mental health outcomes. Qualitative interviews with men in NI who had experienced IPV were used to explore participants’ lived experiences of: IPV within the relationship, formal/informal disclosure, their involvement with support organisations and institutions of the state, mental health impacts, coping, and to determine specific support needs within an NI context. A cross-sectional retrospective qualitative approach was selected to allow for the nuance of a range of personal experiences, which would provide a deeper understanding of these experiences alongside quantitative attitudinal data.

## 2. Materials and Methods

### 2.1. Design and Participants

The ME-IPV Study was designed as a mixed methods study involving (1) an NI wide cross-sectional quantitative survey and (2) a series of qualitative interviews to explore participants’ lived experiences. The current study reports on the qualitative aspect of the study only, which employed Interpretative Phenomenological Analysis (IPA; Smith & Osborn, 2015) to examine the lived experiences of male victims of IPV. IPA was selected as the most appropriate methodology given that it is a well-established qualitative research approach, which focuses on the examination of how people make sense of significant life experiences, thus allowing for an in-depth exploration of the experience and impacts of IPV (Smith & Osborn, 2015). IPA’s combined focus on phenomenology, hermeneutics, and idiography allows for a more detailed, subjective, and interpretative analysis of male accounts of their experiences of IPV, which is especially valuable given the heterogeneous and underrepresented nature of this population (Smith & Osborn, 2015). Moreover, IPA has been utilised across several recent studies examining experiences and impacts of IPV (Giles-Haigh et al., 2026; Sanchez et al., 2025). This study was approved by the Research Ethics Committee, Queens University Belfast (EPS 23_455).

The target population for both the survey and interviews were ≥18 years of age, currently living in NI, male/identified as male, and experienced at least one instance of IPV perpetrated against them. As this topic can be equal parts divisive and stigmatic, especially on social media, recruitment was managed through NI organisations that provide support/services to this population, allowing the research team access to ‘a hidden population within a hidden population’. The research team contacted several NI organisations who agreed to act as stakeholder partners for the purposes of recruitment for this study, including: 2 organisations supporting male IPV victims, 1 organisation supporting men’s health/mental health needs, 1 organisation supporting all victims of IPV, and 1 organisation providing general mental health support. These organisations were provided with digital copies of the recruitment flyer (including a link/QR code for the online survey and contact email address to arrange an interview) and the participant information form to disseminate to their support population. One of the male IPV support organisations facilitated a private Facebook group for NI men with these experiences, which the organisation lead used to further disseminate recruitment materials. It must be noted that this recruitment method did introduce help-seeking bias, as all participants had understood their experiences to be abuse.

Recruitment proceeded until data saturation had been reached, this is defined as the point where no new themes were emerging within the data (Vasileiou et al., 2018). This resulted in a final eligible sample size of 10. Additionally, several other factors informed the final sample size. Firstly, previous literature recommends smaller samples for those employing IPA, typically ranging from 8 to 12 participants (Clarke, 2010; Smith et al., 2022). Secondly, previous research suggests that the optimal sample size is one that fits the parameters and needs of the study (Relja & Ward, 2026). Due to the considerable stigma associated with male IPV experiences in NI, the relatively short recruitment window, and direct recruitment through support organisations, a target sample of 10–15 participants was agreed, and is within the recommended guidelines for a study of this nature (Smith et al., 2022). The mean age was 44.3 years (range 36–62, SD = 6.36), 40% lived in a city, 40% in a town, and 20% in a rural setting. All 10 participants identified as cisgender men who had been in heteronormative relationships with their abusive partners, and all specified that they were no longer in these relationships.

### 2.2. Procedure

All participants (N = 10) consented to participate in semi-structured interviews. Interviews were conducted online using Microsoft Teams or Zoom and typically lasted 60–90 min. Participants were asked a series of open ended questions regarding their experience and associated impacts of IPV (detailed below). Participants were also asked demographic information (i.e., their age and urbanicity) but full demographics were not asked due to the risk of potential identification. A distress protocol was in place (see Supplementary Materials) if a participant was to become distressed, however no incidents occurred to require this. Audio recordings of interviews were transcribed verbatim by the research team and the recordings summarily deleted. Participants names were replaced with ID numbers. Author ES conducted the interviews. ES has several years’ experience in the area of conducting person-centred interviews and research in the area of trauma and mental health across a range of different populations.

### 2.3. Interview Schedule

The interview schedule (see Supplementary Materials) was developed based on the findings of the available IPV related empirical literature (Douglas & Hines, 2011; Bates, 2020a, 2020b; Bates & Carthy, 2020), which examined the experiences and mental health impacts of IPV experienced by males. The interview schedule utilised a series of open ended questions and included (i) age/gender/urbanicity, (ii) context of the relationship, (iii) circumstances surrounding the IPV experience(s), including type, frequency, and duration, (iv) experiences of coping, (v) mental, physical, and social impacts of IPV, (vi) disclosure and help-seeking, (vii) barriers to help-seeking, and (viii) support needs.

### 2.4. Analytic Strategy

Interpretative Phenomenological Analysis (IPA; Smith et al., 1999) was used to analyse the interview transcripts. IPA is a qualitative approach providing detailed examinations of lived experience (Gill, 2014) by utilizing an idiographic focus offering insights into how a given person, in a given context, makes sense of a given phenomenon (Larkin et al., 2006; Smith, 2011). The goal of IPA is to explore how individuals perceive themselves and the world around them and is commonly utilised in qualitative research on sensitive topics, including trauma or mental ill health (Larkin & Thompson, 2012; Hefferon & Gil-Rodriguez, 2011) and IPV (Reynolds & Shepherd, 2011; Shah et al., 2016). This is accomplished by abandoning preconceived notions while analysing the data from a ‘bottom-up’ perspective, employing thematic coding, and generating emergent themes rather than exploring pre-existing hypotheses (Smith et al., 2009; Pietkiewicz & Smith, 2014).

Data was analysed using NVivo 14 (Lumivero, 2023). Authors ES and EM participated in the analysis. Each individual transcript was read and re-read, with specific experiences coded (example: ‘punished for positive live events’). These preliminary codes were reviewed by both ES and EM and consolidated into ‘meta codes’ (example: ‘punished for positive life events’ was added to the ‘IPV type’ meta code). Finally, meta codes were categorised into the main emergent themes (example: the meta code ‘IPV type’ was added to the ‘IPV experience’ theme). As participants tended towards a chronological narrative, usually starting with the beginning of the relationship, the first instance of IPV (or initial abusive behaviours), escalation, the disclosure process, the abuser’s reaction to disclosure, and up to their current state, the research team applied a similar chronological framework in analysis.

Using an IPA framework allowed the team to form interpretive insights from participants’ statements, which formed the basis of preliminary codes. For example, many participants discussed abuse-adjacent behaviour during the early portion of their relationship, applying retrospective reflection that they ‘should have known’ or ‘should have suspected’ there would be abuse based on these behaviours. While none of the participants used the phrase ‘red flags’, the team used this preliminary code, which became the sub-theme ‘early indications of IPV behaviour’.

### 2.5. Reflexivity, Credibility and Rigour

Reflexivity, credibly and rigour were considered throughout all stages of the research process. The lead researcher (ES) is a research psychologist with a professional focus on trauma experiences in specific populations. While this background informed a theoretical sensitivity to the subject matter, ES engaged in ongoing critical reflection to ensure that pre-existing knowledge and assumptions did not unduly shape the interpretative process. ES had no personal experience of IPV or IPV adjacent situations, which was acknowledged as both a potential limitation in terms of experiential understanding and a protective factor against potential over-identification with participant accounts. No members of the research team had any prior relationship with participants, minimising the potential for relational bias in data collection or analysis. To further safeguard analytical rigour, ES engaged in regular reflexive journaling throughout the data analysis process, allowing for transparent documentation of interpretative decisions and emerging assumptions. Peer debriefing was conducted within the research team (ES and EM), providing opportunities for critical challenge and collaborative sense-making. Wider team discussions also took place to discuss findings, researcher reflections and to aid the selection of quotes that attempt to fully reflect the lived experience of the participants (McGlinchey et al., 2021).

## 3. Results

### 3.1. Interview Results

Interview transcripts were analysed using IPA framework (Smith et al., 2009) involving the formation of emergent codes in individual cases, commonality of codes across cases, and formation of meta-codes (Table 1) describing experiences across the sample. For the full code list, see the Supplementary Materials.

#### 3.1.1. Early Indications of IPV Behaviour

Participants described initial ‘red flags’ early on, which were warnings of more abusive behaviour to come. These manifested as small arguments, minor instances of psychological abuse, irrational jealousy, or other behaviour, which participants ascribed to a variety of causes, including ‘pre-wedding nerves’, stress, being pregnant, and physical and/or mental illness. All stated these were small and easily ignored warning signs and some stressed that in the context of a new or blossoming relationship, there is a period of adjustment for both partners as they get to know each other. Many participants expressed regret when retrospectively reflecting on these incidents, interpreting their past behaviour and decisions with the benefit of their current understanding of abuse, stating that they ‘should have known’ or that they ‘missed the signs’, etc.

“That was like, sure, they’re in a bad mood or it was only the one time. And I kind of brushed it off, but a lot of smaller things happened over a long period of time. It wasn’t as if it was a massive, crazy insane incident where you would kind of know straight away that that’s abuse.”

(P002)

#### 3.1.2. Sudden Change in Behaviour

Most described a sudden change in their partner’s behaviour as the ‘red flag’ incidents became worse and/or became a pattern of abusive behaviour. This coincided with major life events: moving in together, getting married, the birth of children, etc. Some theorised that stress or pre-existing mental health problems caused this sudden change, while others speculated their ex-partners had been hiding or minimising their abusive tendencies to reach certain relationship or life milestones. During this period, participants said the home environment was quite unstable and some tried to avoid being at home or hid in personal spaces to avoid touching off abusive events. Participants interpreted this, at the time, as a short-term disruption rather than the transitional phase they later understood it to be. Their reactions to new/worsening abusive behaviour were focused on appeasement, avoidance or trying to understand or correct the issue, though they would later realise no effort would have been successful.

“There was never a bad word between the two of us. We never had an argument. We never fought, I don’t think, until we got married and once we got married then the violence started.”

(P008)

Several participants highlighted they had been unaware of the multimodal nature of IPV and conceptualised ‘domestic abuse’ as purely physical violence. Many expressed they learned about coercive control, psychological, institutional, and financial abuse only after leaving the relationship. This lack of awareness left many blaming themselves for their ex-partner’s behaviour as they had no other explanation for the violence they were experiencing. Participants interpreted this as a failure of a stigmatic society, which categorised IPV solely as physical abuse directed towards women.

“I didn’t even consider it to be domestic violence or a domestic abuse. You know, I had no idea. At that time there were no posters up all around the city saying here’s what domestic abuse is like.”

(P005)

#### 3.1.3. IPV Experiences by Type

Participants’ experiences of IPV fell into 17 broad categories across five domains: psychological/emotional abuse, physical abuse, sexual abuse, coercive control, and institutional abuse. All participants described various forms of psychological/emotional abuse and having false allegations made against them. Here, ‘false allegations’ are defined as accusations made against the participant by their abuser to others, which the participant has stated are untrue. A majority of the sample experienced physical abuse and specific aspects of psychological abuse and coercive control, including manipulation, monitoring/controlling, social isolation, and the involvement of children. Here, psychological abuse covers all non-physical/non-sexual personal abuse intended to adversely impact the mental health of the participant. While coercive control does fall under this definition, it is here considered a separate domain due to the specific intent of control.

#### 3.1.4. Psychological/Emotional Abuse

Experiences of psychological/emotional abuse included shouting, abusive language, gaslighting/manipulation, ‘mind games’, and blaming the participant for situations beyond their control. Specific experiences included manipulation of others against the participant, weaponising children, ‘punishing’ participants for positive life events, harassment by phone/Internet/social media, threats of self-harm and/or suicide, and instances of career sabotage. Participants expressed their interactions with their abusers often left them confused, frightened, or doubting what they knew to be true, and they interpreted this type of abuse as highly damaging and resulting in a significant amount of distress.

“Very quickly I got that cold sense in our marriage that actually anything good in my life, whether it was my family or a new car or a pay rise, I was punished for them, so to speak, so that really took the tone for the rest of our marriage.”

(P001)

Participants described engaging adaptive strategies to try and minimise the damage of psychological abuse or to manage the situation once it got out of hand. They spoke about feeling helpless and/or confused, blaming themselves, being uncertain of their own words and actions, or who they could rely on. Notably, one participant stated he would have preferred physical violence over psychological abuse as he felt he could better heal from physical injuries over the complex process of healing from psychological abuse.

“I honestly feel I would have preferred if my partner would have beat me every day because I could physically recover from those kind of things and have left my scars, but I have been completely destroyed and my mental health has suffered so much.”

(P002)

#### 3.1.5. Physical Abuse

Physical abuse covered a range of actions, including being hit, slapped, punched, kicked, shoulder-checked/shoved, threatened with knives, hammers, and other improvised weapons, cut, scratched with nails, and having objects thrown at them.

“She grabs my neck with her hands, long, relatively long nails, and just starts clawing at my neck with her hands. She drew some blood on my face as well, and then just starts whacking me, just slapping me as hard as she possibly could across the face.”

(P005)

“Threw bottles at me, threw tins of beer at me, full tins of beer. She cut my nose. She caught me, she hit me in the ears, split me ear.”

(P008)

Several spoke about trying to protect themselves without taking retaliatory action by backing themselves into corners or getting behind a closed/locked door. Importantly, participants described physical disparities between themselves and their ex-partners, and the fear that ‘no one would believe’ their ex-partner had physically abused them. Some stated that they expected a certain amount of gendered violence, seeming to imply either they did not consider specific types of physical violence to be ‘actual’ violence (slapped but not punched, etc.) or they were acknowledging societal acceptance of physical violence from women as inherently less threatening. This interpretation tied into their identities as men; they did not want to hurt a woman by fighting back or defending themselves but in doing so, had to allow themselves to be hurt and placed into an identity of victimhood.

“Like she used to slap me and push me and but, you kind of expect that from women to an extent.”

(P003)

“I didn’t go to my GP to report it because it’s expected, if your girlfriend’s angry at you, she might push you or slap you.”

(P005)

#### 3.1.6. Sexual Abuse

Sexual abuse included sexual contact without consent and instances of reproductive coercion. UK law specifies that as rape can only be perpetrated by an individual with a penis (Sexual Offences Act 2003; Ministry of Justice, 2003), women cannot be charged with/prosecuted for rape, only lesser charges of sexual assault. Reproductive coercion concerned repeated instances of psychological abuse when the participant asked to use a condom and misuse of contraceptives to conceive a child against the participant’s wishes. Discussion of sexual abuse was distressing, as participants again interpreted their experiences through the lens of their male identities in light of the narrow UK legal definition of rape and societal stigma against the reality of male victimhood in sexual assault.

“She came back early, drunk, and I was asleep in bed. And like basically raped me, like I know you can’t really say rape because you know, but at the same time, I kept telling her, ‘No, don’t do this!’.”

(P005)

#### 3.1.7. Coercive Control

Coercive control included monitoring or controlling behaviour (including smart devices), socially isolating the participant from friends/family, financial abuse, and weaponizing the participant’s relationship with their children. Some stated they were made to account for their whereabouts at all times, including during the workday. They described their lives as being controlled both directly and indirectly, frequently being told where they were permitted to go, and with whom they could communicate. Participants interpreted this as their own lives occurring outside of their active control where ‘keeping the peace’ became more important than retaining personal agency.

“She then took our keys off me, she says, ‘You’re not allowed anywhere. You’re staying here.’.”

(P010)

Social isolation was accomplished through psychological abuse and manipulation when participants tried to participate in social relationships, control of contact with others, and seeking to ‘poison’ relationships with family/friends, manipulating others, and harassment and/or abuse of others to drive them away from participants. These behaviours succeeded in cutting participants off from their support networks, depriving them of individuals who might have helped them escape the abuse, and reinforcing the idea the ex-partner was the only person the participant could trust/rely on. When reflecting on disclosure, many participants understood that social isolation had negatively impacted on this process.

“She would have told me that people we knew were talking about me and that really isolated me from those people and gave me a deep sense of insecurity that, who did I have that I could go to, or who did I have that I could confide in?”

(P001)

Several reported experiencing financial abuse, specifically the ex-partner either being unemployed or not engaging in realistic spending behaviours, resulting in participants putting a majority of funds towards household/child expenses. Participants spoke about wanting to leave but being ‘trapped’ by financial obligations to the household and having no funds for an exit strategy. Some participants interpreted these experiences as being tied to their identity as men and the need to be financially stable as a ‘provider’ for their children or family. Financial control dovetailed with the ex-partner involving children in the abuse. This included psychological and verbal abuse of children if they agreed or sympathised with participants, withholding access to the children, alleged physical abuse/neglect by the ex-partner, and in one case, illegally removing the children from the UK.

“That’s when she started getting the daughter in fold by getting the daughter to ask me to leave because her and her mum would have a better relationship.”

(P007)

“She started on the child, ‘How dare you! You treat me like dirt!’ And then she turned around and says, ‘It’s your daddy, it’s his fault!.”

(P010)

#### 3.1.8. Institutional Abuse

Institutional abuse covered weaponisation of institutions of the state, which included reports of harassment through solicitors, manipulation of institutions to control the participant, retaliatory/punitive use of the courts, police, and social services, and perjury. Importantly, institutional abuse contributed to financial abuse, as participants did not have access to public legal funds and had to pay for legal involvement. Many described repeated delay tactics, and all participants described issues with the courts/legal system not recognising them as IPV victims.

It is important to note the difference between participants’ adversarial perceptions of the institutions of state and the legal reality of the NI system. Specifically, there are significant differences in the functions, processes, and remit of criminal courts compared to civil courts/family proceedings. ‘Respondents’ in family court who are victims of abuse have the ability to access legal assistance without the need for a financial eligibility test (Department of Justice, 2022) via the ‘domestic abuse waiver’. This has caused confusion, as those involved with criminal proceedings would continue to engage with the legal aid system as normal. This waiver was originally implemented to prevent abusers from utilising the family courts to further perpetrate abuse, and the NI Department of Justice undertook public consultation on changes to this system in 2024.

“She withdrew them all, usually after it cost me £2000 in representation fees at various hearings that have been adjourned by her legal counsel that were getting legal aid and could adjourn all day long and just play with government money to do it.”

(P004)

All participants reported their ex-partners had made false statements or allegations against them to friends/family, police, courts, and social services in an attempt to manipulate, control, or isolate them. Allegations included accusing the participant of IPV, physical/sexual abuse of children, child neglect, stalking/harassment, alcohol/substance misuse, and being unfit due to mental health issues. Participants spoke about their distress being compounded by the stress of false accusations and spending considerable time/money attempting to clear their names. These experiences caused significant distress, as participants explored the difference between their perception of their identity as a ‘good’ or moral person, and the version of themselves that their abuser created through these statements.

“I’ve not done nothing and that’s exactly what it was. He [police officer] said, ‘I’ve got no times. I’ve got no date,’ he said, ‘These are just random allegations,’ I said, ‘Well, why are you even listening to it then?’.”

(P006)

“Since I have moved into the flat here, I’ve had 79 police visits. I have been arrested here twice. She had said that I was making false allegations to police, and was harassing her. And turns out that when police did their investigation, that her brother who lives in England was making them on her behalf.”

(P010)

#### 3.1.9. Coping

Some participants described spending more time in the workplace, escaping the situation/room/house, and exercise as coping strategies. Others discussed either having no coping strategies, or strategies that were unhealthy/harmful. These included shouting while alone, isolation, maladaptive eating habits, self-harm, alcohol misuse, and internalising or ‘pushing down’ negative emotions. Many who shared unhealthy coping strategies stated they understood these methods were potentially harmful but they did provide some measure of relief. There was a common thread among participants of retrospectively identifying their behaviours as coping behaviours once asked about these strategies, allowing them to critically interpret these behaviours.

“I drank a lot more than I should have in the immediate short term. So it was almost a kind of coping mechanism and not a very good one. I can really see how someone could spiral into alcoholism with that because it’s just, it numbs the sadness, the depression and helped me kind of nod off.”

(P002)

“So in terms of like coping mechanisms, I guess ignore it, is the big one. You know bottle it up, push it down, forget about it.”

(P003)

#### 3.1.10. Physical Impacts

Participants described a range of physical impacts, including cognitive impairment (‘brain fog’), sleep disturbances, and a range of medical conditions brought on or exacerbated by their experiences. A few discussed no longer being able to exercise due to stress-related decrease in motivation and several reported an associated decline in hygiene motivation and healthy eating habits. Importantly, several of these physical impacts have known associations, i.e., sleep disturbances are well-established contributors to overall poorer physical health, and adverse changes in diet/exercise can lead to a range of cardiovascular outcomes. It must be noted that while there is no clear causal link between physical symptomology and/or condition exacerbation and participants’ IPV experiences, participants interpreted these as related.

“I think something that I experience a lot is brain fog, it generally feels like there’s just, there’s a cloud in the brain at all times and I can’t kind of think properly, remember things properly or just string a sentence together at times.”

(P002)

#### 3.1.11. Psychological Impacts

Participants described a range of psychological/emotional impacts, including fear of their ex-partner, grief from family dissolution, hopelessness, and feelings of injustice. Some discussed how their senses of personal security and trust have been damaged, describing hypervigilant behaviour consistent with post-traumatic symptomology. Others described ‘not living, just surviving’, feeling that they had lost everything that was previously meaningful, and a significant loss of agency and self-worth. Several spoke about mental/emotional ‘paralysis’, a feeling of not being able to make decisions, start/finish tasks, meet deadlines, or make social plans, with some having taken a leave of absence from employment.

“In the long term, I have experienced mental health problems, depression and anxiety. These all have been down to, looking back on it now, with time and space to reflect on it, I honestly think that it is solely down to the abuse.”

(P010)

Most had managed distress and mental illness symptomology under the care of their GP and various types of counselling/care. Importantly, participants employed a duel interpretation of mental health impacts, referring to both formal diagnoses (anxiety, depression, and PTSD) and symptomology as separate and distinct phenomena. In a few cases, participants described pre-existing mental health issues, which had been exacerbated by the stress of their IPV experiences. One participant shared his insight on the nature of depression and the stigmatic beliefs he had held.

“I was a strong believer that depression didn’t exist years ago. I’m like, ‘You’re not depressed. You’re just being weak. You need to find a way out of it. OK, someone died. They’re not going to come back. You need to continue with your life.’ I was taken for a reality check. I had to go on antidepressants [and] beta blockers.”

(P009)

#### 3.1.12. Suicidality

Multiple participants discussed feeling suicidal, formulating a plan, and making one or more attempts to take their own life. For them, suicide was described as an escape from the pain of past IPV experiences but also from the certainty of continuing abuse from their ex-partner and weaponised institutions. These experiences were described alongside feelings of hopelessness, loss of agency, and depression. All participants discussing suicidality were asked about any current ideation and fortunately, all stated they had been able to seek help through a variety of support measures. Experiences of suicidality were interpreted as a logical, though upsetting outcome of abuse, distress, and stress.

“And I did get a point where I did tell him [GP], I said, ‘Look, I feel like just putting a rope up and that’s it.’.”

(P008)

“The abuse had got so bad that I actually had a rope tied to hang myself that day. And I knew my eldest girl […] I knew that she would be the one coming home from school to find [me] and that’s the thing that pulled me back.”

(P010)

As with depression, two participants said their experiences have brought an understanding of suicidality, whereas before they had held stigmatic beliefs.

“I do have an understanding now why people do it. I can see the line that people take and justify in their own mind why they do it. Before, I used to think, ‘You inconsiderate bastard. What the f*** is wrong with you?’ But with everything that’s going on, had I have been a weaker mental person, who knows?”

(P006)

“My whole life […] I’ve always believed suicide is a very selfish act. I’ve had people try to explain how, ‘You don’t understand, it’s the chemical reactions or imbalances that make people do it,’ and I’d think no, they’re not, it’s selfish. And then there was a day I walked into the kitchen and I seen the Stanley knife.”

(P009)

#### 3.1.13. Social Impacts

Participants impacts on how they perceived themselves on a social level, including social isolation. For some, this was the aftermath of isolation being used against them, but some disclosed they had self-isolated due to poor mental health. Many had trouble dating and/or starting new relationships, romantic or otherwise. Some described damage to their reputations and employment prospects alongside feeling ‘criminalised’ due to their ex-partners’ false allegations, leaving them feeling twice-victimised. As NI is not a large country, there was concern the damage to their reputations would be lasting, if not permanent. Participants interpreted this as another facet of abuse rather than an outcome.

“When this all first came out […] I just didn’t feel easy even though I was proved innocent, but still didn’t feel easy walking about in my own home town. So just basically had to move away from my family and all.”

(P007)

#### 3.1.14. Experiences of Disclosure

Experiences of disclosure to family members were mixed, with some family not believing them or siding with their abuser, while others immediately believed/supported participants. Experiences of disclosure to friends were similar, ranging from disbelief to wholehearted support. For some, disclosure to the police resulted in positive outcomes (non-molestation orders, ex-partner removed from shared accommodation, arrest and investigation, etc.). Participants interpret their experiences of disclosure on personal and societal levels; they ascribed personal motives to individual reactions of family and friends and societal influences/biases to the reactions of institutions of the state.

Many participants found the police dismissive of their experiences. They described reporting IPV incidents only to later find those incidents were not recorded, being told they were just ‘being dramatic’ or to ‘man up’, or accused of lying. Participants described being told by either police or the PPS, without explanation, there would be no investigation into their reports. This was a very detrimental experience, and many explained how they overcame significant fear and distress to disclose, only to be met with apathy or outright hostility. In some cases, participants’ first disclosure was made during an IPV incident where they had contacted police for their own safety. Unfortunately, several of these incidents resulted in an immediate counter-allegation and the participant was arrested after phoning for help.

“The police were hopeless. I mean, they literally just looked at me, they saw I’m 6’. She’s 5’2 and they were just like, ‘You’re claiming she’s done this? Get over yourself, mate. Like you should be able to handle yourself.’.”

(P005)

“The police officer that dealt with it was an absolute shambles. He stood in the inquiry office, he says, ‘Catch yourself on, Mr. [P010]. Do you expect me to believe that a big feller like you was abused by your wife?’ And I just turned and walked out.”

(P010)

#### 3.1.15. Barriers to Help-Seeking

Participants described barriers, which fell largely into the three main categories: logistic, stigmatic, and trust barriers.

Logistic barriers included the lack of a clear care pathway and an initial lack of awareness that their experiences constituted IPV. Participants described searching online for information around disclosure and support but finding very little and post-disclosure, encountering poor signposting of support resources. This led to feelings of doubt and reinforced the stigmatic belief they had to ‘man up’ and keep silent as there was no one who could help them. Several stated they did not disclose or seek support at first because they thought of IPV only as physical abuse.

“I didn’t even know where to where to turn. I’ve Googled loads of stuff and people have said, ‘You need to speak to this. You need to speak to that,’ and you phone them up and you’re not suitable or it’s not something they deal with and then they pass you on to another number.”

(P006)

Stigmatic barriers included fear/anxiety around disclosing, fear of not being believed, of social, legal, or reputational consequences, that abuse would worsen if they disclosed, and feelings of self-doubt/stigma around their experiences. Gender stereotypes and the public perception of IPV victims as women also substantially contributed to participants hesitance to disclose/seek support, particularly the belief that disclosure would cast suspicion on them. These stigmatic barriers were reinforced by social media, where gender discourse complicates issues of IPV disclosure. However, participants interpreted their experiences through a lens of initial self-stigma, acknowledging that it was only after they sought and received help, that their outlook on disclosure and help-seeking evolved to its current state.

“I knew I wouldn’t be believed and I’m still not believed to this day. I almost feel like there’s a lot of shame there and yeah, maybe the biggest barrier was my own head and maybe there weren’t many physical barriers. I was just thinking that I won’t be believed and I won’t be given fair treatment.”

(P002)

Trust-based barriers were the least prevalent but focused on lack of trust and faith in the court/legal system and the belief that disclosing would only make the situation worse. Participants felt that, given everything that had happened to them and hearing the experiences of other men, they did not trust the mechanisms of state. When discussing these barriers, several participants shared their initial misgivings had been correct. These interpretations were most common in participants who had adversarial experiences with institutions, and it must be noted that some participants did report feeling justified in trusting the system.

“Everything’s against the man and I’m not a sexist in any way but everything has been piled against me for the last three years and I’ve been fighting the system that’s not interested in men.”

(P006)

#### 3.1.16. Experiences of Support

Speaking of the support they received from family members, a few participants clarified that while their family supported them in general, some failed to understand the significant toll the abuse had taken and others eventually ‘came around’ after not believing them. This support was not without a price, as several described their ex-partner turning abusive towards their family members. Several expressed how grateful they felt when, after having been socially isolated from their friends, these friends turned supportive after disclosure. Participants interpreted support from family/friends as a validation of themselves as a person, that supports had been able to ‘see through’ lies or manipulation to the ‘real’ truth of their identity as a victim.

“I’m really lucky I met my now wife because she’s been incredible throughout. She’s just, like endured a whole pile of s*** that she shouldn’t really have had to endured.”

(P005)

“The family was in court with me throughout the two days. So they were very supportive. They knew right away that’s it’s all lies.”

(P007)

Most discussed their experiences with formal counselling/therapy, with opinions being generally positive. Participants cited how counselling helped them process their experiences, learn or improve coping methods, and reduce distress. One criticism, however, was that though it was helpful in processing immediate distress, there was little a counsellor/therapist could do to address the root cause of participants’ ongoing abusive situations. Many spoke highly of informal support, namely group counselling or peer support groups. Through these groups, participants felt they were not alone and their experiences were validated by meeting other men who had gone through similar. Some groups facilitated social events, allowing participants to socialise without fear of stigma, fostering stronger support networks.

“I’ve done some therapy sessions and in-person sessions. Going into the trauma, different things happening and also then down in the face-to-face where you learn boundaries.”

(P009)

“It’s peer support, so everybody has been through a different situation, and everybody’s kind of, feeding into, you know, ‘This helped me. This helped me, try here,’.”

(P010)

#### 3.1.17. Post-IPV Recovery

Participants discussed recovery after leaving their abusive relationships and accessing support. This focused on regaining lost self-worth/confidence, improving physical/mental health, feeling secure to enter new relationships, and engaging socially. While nearly all interview participants had children with their ex-partners and will have to engage with them until the children are adults, some expressed relationships with their children were improving as the children aged and better adapted to the new family dynamic. Participants tended to interpret the series of experiences as a linear ‘journey’ with the (assumed) end-point being their abuser unwilling or unable to continue victimising them, resulting in an end to their distress.

“Many of my days I’m still in that paralysis thing. Things that I need to do, I just can’t do them. I feel like I’m lazy. But I know that I’m not. I know that I am almost in a protective kind of mode where I’m just resting everything. I have to let that happen, I think.”

(P001)

“It’s obviously been incredibly challenging and draining emotionally, but I can happily say that, yeah, I now feel like I’ve built a life that I’m actually proud of and I’m remarried. We’ve just had our first child together.”

(P005)

## 4. Discussion

ME-IPV interview participants described experiencing multiple forms of IPV, with coercive control, psychological, and institutional abuse being highly prevalent. Several participants discussed fear of their abusive partner due to coercive control, including fear of physical harm and fear of the consequences of their partner’s actions, which is concordant with previous findings (Powney & Graham-Kevan, 2022). Psychological abuse caused considerable distress to participants, aligning with previous findings in both men and women (Dokkedahl et al., 2022; Dumont et al., 2022). False allegations and institutional abuse, which persisted after the dissolution of the relationship, are known to be a particularly successful abuse tactic (Bates et al., 2022a), which can result in significant distress, as was evident here. In this sample, non-physical IPV was responsible for a majority of the trauma experienced by participants, interpreted through the lens of their identities as men, members of NI society, and ultimately as victims.

Participants’ experiences of IPV had detrimental effects on mental health. This finding validates previous research on the extensive psychological impact of IPV on men (Hines, 2007; Lagdon et al., 2014, 2023; Scott-Storey et al., 2023; Macassa et al., 2023; Landa-Blanco & Mejía Sánchez, 2025). It is important to note that in this help-seeking sample, participants understanding and interpretation of poor mental health/distress symptomology may have been informed by the help they received. That is to say, they have retrospectively identified these issues due to a new/better sense of mental health awareness. Psychological and cognitive symptomology (including ‘brain fog’ and executive functioning deficits) are well-associated with IPV experiences, however, the possibility that these were partially due to IPV-related neurological injuries (E. Valera & Kucyi, 2017; E. M. Valera et al., 2019; E. M. Valera, 2018; Daugherty et al., 2021, 2024) should also be considered.

Some participants had dealt with suicidal ideation and attempts due to their experiences. It is well established that men are more likely to complete suicide and less likely to report suicidality (Scourfield & Evans, 2015), with higher self-identification with traditional male behavioural norms associated with increased suicide risk (Griffin et al., 2022). For men who have experienced IPV, this risk may be compounded by further feelings of emasculation due to the abuse, especially stigma related to help-seeking (Macassa et al., 2025). Suicidality is also highly stigmatised in NI (O’Neill & O’Connor, 2020; O’Neill & Devine, 2022), which could force male IPV victims into a situation where reluctance to disclose (both abuse and/or suicidal ideation) could result in increased risk of completed suicide.

Participants discussed high levels of trauma exposure, both relating to the abuse and unrelated trauma, and it must be noted that on a population level, NI has a higher rate of cumulative trauma exposure when compared to similar countries (Ferry et al., 2014; Redican et al., 2022). While cumulative trauma exposure is associated with an increased risk of post-traumatic distress/PTSD, trauma exposure does not necessarily result in traumatisation (Robinson et al., 2022; Spikol et al., 2024b). However, the complex effects of cumulative trauma should be taken into consideration when designing trauma-informed initiatives for men with the experience of IPV. Participants utilised a range of both adaptive and maladaptive coping strategies in response to their experiences, though the study was not able to measure the efficacy of these. Future research should explore the impact of coping strategies on resilience in this population.

Participants described disclosing to family/friends with mixed results of belief and support, though experiences were generally positive and many participants felt supported. In disclosing to the police, a few participants had positive experiences, but a majority reported negative interactions in line with previous research (Lockwood & Prohaska, 2015; Walker et al., 2020; Ewin, 2022). Negative post-disclosure interactions with the institutions of state (courts, child/family services, police, legal representatives, etc.) were again in-line with previous findings (Macassa et al., 2025) and pose a public health risk; men with societal expectations of poor post-disclosure treatment may decide not to disclose or seek help.

When discussing help-seeking and any literal or perceived barriers to care, participant responses broke down into three main categories: logistic barriers, stigmatic barriers, and trust-related barriers. These categories mirror the internal/external/systems model for barriers-to-care, describing personal, extra-personal, and institutional stigmas, which contribute to delay in care seeking (Bates et al., 2022b; Lysova & Dim, 2025). However, this model could also lay the groundwork for a tripartite awareness campaign designed to bolster help-seeking by decreasing all three categories of stigma.

Participants’ experiences of informal support were mostly positive and participants spoke highly of their interactions with group/peer support, including social media. Informal avenues of support should not be overlooked when considering multidisciplinary initiatives for post-IPV care. These spaces allow male IPV victims to ‘relax’ among others with similar experiences, as well as sharing advice, information, and comradery. As for formal support, while many participants found this beneficial in relieving distress/symptoms of mental ill health, processing their experiences, and learning cognitive strategies, several participants criticised the long-term efficacy of counselling in the face of ongoing abuse by their ex-partner. This remains a crucial issue in post-IPV care for men, as formal support can only address adaptive/reactive symptomology and so long as the abuser is engaged in abuse, cannot address the root cause of the distress.

### 4.1. Impact and Implications

The ME-IPV study follows McGlinchey et al.’s (2023) systematic review of literature summarising the global prevalence of IPV perpetrated against men/boys and the mental health impact of these experiences, which called for direct research of this public health issue within NI. The abuse perpetrated against participants has caused them considerable distress and let to multiple adverse health and mental health outcomes. Additionally, these events have affected their children, family/friends/others, and led to often adversarial experiences with police, courts, and institutions of state. Participants also experienced harmful stereotypes, a lack of awareness, and stigmatic beliefs from various sectors of NI society when making the choice to disclose. Due to the impact of the cost-of-living crisis on third sector funding across the UK (Association of Charitable Foundations, 2024; Northern Ireland Affairs Committee, 2024; National Council for Voluntary Organisations, 2025), charities/support organisations in NI frequently lack the resources to provide sufficient support for men who have experienced IPV.

The findings from the ME-IPV study should be taken as an evidence-based call to action for change in NI across research, practice, and policy. Future research should prioritise sub-populations, including LGBTQ+ individuals (specifically transgender men and men in same-sex relationships), ethnic/cultural minorities, and older men (+65). Further studies should concentrate on psychological mechanisms associated with adverse outcomes based on IPV type exposure and the impact of coping strategies as a potential mediator. While no participant espoused these views, this population may be vulnerable to online radicalisation based on experiences of stigma or gender bias/discrimination after disclosure; future research should explore the extent of this issue in NI.

When funding allows, charities/support organisations who service men with the experience of IPV should invest in expanding their remit, collaboration, and outward-facing education/training literature with an eye towards awareness and reducing stigma. Third-sector organisations should signpost clear eligibility requirements for service users. These organisations who support individuals who have experienced IPV (regardless of gender) should consider forming a task force or executive advisory group to foster collaboration and contribution towards a united front against abuse in NI, including public awareness campaigns.

NI policy should consider male victims of IPV during planning and execution stages through adaptation of existing legal pathways, appointment of a liaison, and through governmental support for IPV awareness and stigma reduction campaigns. Additionally, policymakers should consider the creation of an executive-level committee at the NI government level for all matters of NI law/policy involving IPV, including third sector or independent consultation.

### 4.2. Strengths and Limitations

There were several notable strengths associated with the ME-IPV Study. This is, to date, the first exploration into the physical/mental health impacts and experiences of IPV in NI men. The mixed-methods nature of this study allowed for a more nuanced picture of this population by contextualising statistical results with participants’ experiences in their own words. Additionally, the research team worked directly with local stakeholder charities/support organisations to recruit participants from a hard-to-reach population.

The findings here must be taken in light of limitations of the study. Due to the stigmatic nature of disclosure and the hidden population status of participants, limited sociodemographic information was collected from interview participants to avoid potential identification. As is true with all self-report data, findings here may feature social desirability bias (van de Mortel, 2008), and retrospective recollections may feature recall bias (Althubaiti, 2016). Recruitment was limited to individuals who had previously engaged with stakeholder/charity organisations, thus participants here represent a help-seeking sample and therefore introduce a help-seeking bias. The experiences of male/male identifying members of the LGBTQIA+ community and ethnic minorities could not be explored due to the low sample size and homogeneity of the sample, thus the findings here reflect only the experiences of cisgender men in heterosexual relationships. Finally, as there is no known profile of NI men who have experienced IPV and due to the small sample size, sample homogeneity, and the above points, findings cannot be taken as representative and cannot be generalised to the superordinate population.

### 4.3. Conclusions

The findings here point to male experiences of IPV in NI being a significant public health issue, which warrants immediate attention. These results validate the prior literature associating male IPV victimisation with a range of adverse health, mental health, and wellbeing outcomes. Additionally, they add to the body of evidence documenting the significant self/societal stigma and barriers to disclosure, care and help-seeking that male victims face. The impetus is on the academic, statutory, and third sectors of NI to act in supporting men with these experiences. Despite the adverse physical and psychological outcomes described in this study, change, awareness, and reform are possible.

## Figures and Tables

**Table 1:** Theme framework and meta-codes.

Themes
IPV Experiences Early indications of IPV behaviour Sudden change in behaviour IPV experiences by type ○ Psychological/emotional abuse ○ Physical abuse ○ Sexual abuse ○ Coercive control ○ Institutional abuse
Coping
IPV Impact Physical impacts Psychological impacts Suicidality Social impacts
Experiences of Disclosure
Barriers to Help-Seeking
Experiences of Support
Post-IPV Recovery and Meaning Making

## Data Availability

The data presented in this study are available on request from the corresponding author to preserve the privacy of participants.

## Supplementary Materials

The following supporting information can be downloaded at: https://www.mdpi.com/article/10.3390/bs16061007/s1, Distress Protocol, Interview Schedule, Full Code List. Draucker et al. (2009) was cited in the Supplementary Materials.
