# Supplementary material for: Experiences and Impacts of Intimate Partner Violence Against Men in Northern Ireland: Qualitative Findings from the Male Experiences of Intimate Partner Violence Study
Source: Behav Sci (Basel). 2026 Jun 16;16(6):1007. doi: 10.3390/bs16061007 (PMC13296042; doi:10.3390/bs16061007)
Supplement: Supplementary file 1 [file behavsci-16-01007-s001.zip › Distress Protocol.pdf]

# Distress protocol

**Participant Distress protocol**

*(Adapted from: Draucker, Martsolf and Poole (2009) Developing Distress Protocols for research on Sensitive Topics. Archives of Psychiatric Nursing 23 (5) pp 343-350)*

**Distress**

- A participant indicates that they are experiencing stress or emotional distress ***OR***
- A participant exhibits behaviours suggestive that they are experiencing stress or distress, for example crying, shaking, agitation.

**Stage 1 response**

- The researcher stops the interview.
- The participant is offered immediate support by the researcher through assessing their mental status, e.g.:
    - "Can you tell me what thoughts you're having?"
    - "Can you tell me what you're feeling at the moment?"
    - "Do you feel safe at the moment?"

**Review**

- Ask if the participant feels able to carry on:
    - If so, resume the interview.
    - If the participant does not wish to carry on, go to stage 2 response.

**Stage 2 response**

- Discontinue the interview and cease recording if possible.
- Offer immediate support if appropriate, e.g. advising mental health first aid skills, breathing techniques, etc.
- Encourage the participant to seek support from their usual contact points – their GP, a psychologist, or their mental health team if applicable.
- Offer, with participant consent, for the researcher to contact an agreed professional within their support system.

**Follow-up response**

- Encourage the participant to seek support if they experience increasing levels of distress in the hours and/or days post interview.
- Ensure the participant has an information sheet with the contact information for having their data removed or any further questions about the study.
