# Supplementary material for: Experiences and Impacts of Intimate Partner Violence Against Men in Northern Ireland: Qualitative Findings from the Male Experiences of Intimate Partner Violence Study
Source: Behav Sci (Basel). 2026 Jun 16;16(6):1007. doi: 10.3390/bs16061007 (PMC13296042; doi:10.3390/bs16061007)
Supplement: Supplementary file 1 [file behavsci-16-01007-s001.zip › Full Code List.pdf]

Report Themes	Original Codes
IPV Experiences	
Early indications of IPV behaviour	• Initial red flags
Sudden change in behaviour	• Sudden change in behaviour
IPV experiences by type	
Psychological/emotional abuse	• Psychological or emotional<br>• Manipulation (others)<br>• Involving children<br>• Parental alienation<br>• Punished for positive events<br>• Harassment (all types)<br>• Self-harm & suicide threats<br>• Career sabotage
Physical abuse	• Physical (general)<br>• Sleep deprivation
Sexual abuse	• Sexual assault<br>• Reproductive coercion
Coercive control	• Monitoring or controlling behaviour<br>• Social isolation (of participant)<br>• Involving children<br>• Financial<br>• Parental alienation
Institutional abuse	• Institutional (general)<br>• False allegations
Coping	• Adaptive or protective behaviours<br>• Escape<br>• Exercise<br>• Ignore, block, or push down<br>• Unhealthy coping
IPV Impact	
Physical impacts	• Medical condition<br>• Cognitive impairment<br>• Nightmares<br>• Sleep disturbances (all other)
Psychological impacts	• Emotional (general)<br>• Fear, grief, or hopelessness<br>• Hypervigilance<br>• Injustice (emotional)<br>• Loss of agency<br>• Mental health (general)<br>• Paralysis<br>• Self-harm<br>• Self worth<br>• Trust issues
Suicidality	• Suicidal ideation<br>• Suicide attempt<br>• Understanding suicidality
Social impacts	• Criminalised<br>• Dating<br>• Employment<br>• Reputation

	• Social isolation
Experiences of Disclosure	• Didn't disclose<br>• Evidence & documentation<br>• Family<br>• Friends<br>• GP<br>• Others<br>• Not believed<br>• Police
Barriers to Help-Seeking	• Logistic<br>o Lack of care pathway<br>o Lack of IPV awareness<br>• Stigmatic<br>o Fear or anxiety<br>o Gender bias<br>o Might not be believed<br>o Public IPV awareness<br>o Self-doubt<br>o Social media<br>o Stigma & self-stigma<br>• Trust<br>o Institutional<br>o Risk of legal consequences
Experiences of Support	• Counselling<br>• Family<br>• Friends<br>• Group or peer<br>• Local (NI charities/orgs)
Post-IPV Recovery & Meaning Making	• Post-IPV Recovery<br>• Meaning making
Future Recommendations	• Awareness<br>• Collaboration<br>• Dedicated care pathway<br>• Education<br>• Judicial reform<br>• Legal accountability<br>• Support funding<br>• ME-IPV experience<br>o Motivation<br>▪ Change<br>▪ Help others<br>▪ Help own children
