# Supplementary material for: Experiences and Impacts of Intimate Partner Violence Against Men in Northern Ireland: Qualitative Findings from the Male Experiences of Intimate Partner Violence Study
Source: Behav Sci (Basel). 2026 Jun 16;16(6):1007. doi: 10.3390/bs16061007 (PMC13296042; doi:10.3390/bs16061007)
Supplement: Supplementary file 1 [file behavsci-16-01007-s001.zip › Interview Schedule.pdf]

Qualitative Interview Schedule

Introductory statement: Thank you for agreeing to take part in this interview. As you know, we're conducting this study because we're interested in the experiences of men in Northern Ireland who have had the experience of intimate partner violence perpetrated against them. In particular, the questions I'll be asking you today focus on what it was like for you being in a relationship where you had those experiences, the impact that they had on you, any support that you had, and what you believe needs to change in the future to help support yourself and other men who've had these experiences.

Before we start, I want to let you know that you can stop the interview at any time and for any reason. If you begin to feel stressed or distressed, we can either pause the interview for a few moments or end it entirely if you don't wish to continue. If you change your mind about participating and wish to withdraw from the study, simply inform us and any information that you've provided will be deleted/destroyed. If you choose to withdraw after the interview is completed, you will have 1 month to let us know, otherwise analyses including your data will have begun. At the end of the interview, I'll also be providing you with a list of support resources to have on-hand in case you need them.

This interview is completely confidential. I will be recording the audio, transcribing the recording to a text document, and then deleting the audio. Any information which could potentially identify you will be redacted and your transcript will be associated with an ID number to ensure your confidentiality. Only myself and the research team will have access to the pseudonymized transcript. However, I need to let you know that if you tell me that you're in serious risk of harming yourself or someone else, or if you give me specific details of a crime which has not yet been reported, regardless of perpetrator, I must break confidentiality and give this information to authorities to ensure your safety and the safety of others.

I'll be asking you several questions. Feel free to think before you answer and take as much time as you need. Depending on your answers, I may ask follow-up questions to make sure I have a good understanding. If you're comfortable and ready, we can begin.

DEMOGRAPHICS

Key sociodemographics will be asked in a short Qualtrics survey (see Appendix 15) sent to the participant after they have signed consent, to be completed prior to the interview.

Questions	Probes
Gender, age, urbanicity	N/A

CONTEXT, BACKGROUND, AND CIRCUMSTANCES OF IPV

Questions	Probes
Intimate partner violence can take the form of physical violence, sexual violence, stalking, and psychological aggression, including coercive or controlling tactics. Please keep these examples in mind for the next several questions.	N/A

Can you tell me about the relationship you were in when you experienced intimate partner violence?	• How long ago was this?<br>• How long had the relationship been going on when you first experienced IPV?<br>• Are you still in this relationship?
How would you describe the types of IPV you experienced?	• Did this happen more than once?<br>• How frequently did this happen?
Were these experience always present in the relationship?	• When did they start ?<br>• Did they occur at any particular times?
Was this the first relationship where you had these experiences?	• If not first relationship, then further probing re that relationship in line with the above questions. Max of two prior relationships

COPING

Questions	Probes
How did you find yourself coping with your experiences?	• Was/were your coping method(s) helpful?<br>• Was/were your coping method(s) harmful (alcohol/substance misuse)?<br>• Was there anything that was especially helpful in your coping process?

DISCLOSURE, SUPPORT, HELP-SEEKING, AND BARRIERS

Questions	Probes
Did you disclose your experiences to anyone?	• Did you disclose your experiences to any authorities (PSNI, school, employer, GP/mental health professional)?<br>• Did you disclose your experiences to family, friends, coworkers, or spiritual leaders? [Clarify who]<br>• Why did you tell that person / those people?
(If disclosure) What kind of support did you receive?	• Who provided this support?<br>• Was this support helpful and how did it impact on you?<br>• Are there any type(s) of support you now wish that you'd had?
(If no disclosure) What prevented you from disclosing your experiences?	• Have you sought support for the impact of your experiences without disclosure?

What barriers did you experience which may have hindered you in disclosing your experiences or seeking support?	• Was there anything in particular that prevented you from disclosing?<br>• Was there anything in particular that prevented you from seeking support?

IMPACTS OF IPV & FUTURE SUPPORT NEEDS

Questions	Probes
What would you say have been the short-term impacts of your experiences on your physical health, mental health, and/or wellbeing?	• Have you been able to access support for dealing with these impacts?<br>• Do you feel like you got the support that you needed?<br>• What extra support would have helped you to cope with this experience?
What would you say have been the long-term impacts of your experiences on your physical health, mental health, and/or wellbeing?	• Have you been able to access support for dealing with these impacts?<br>• Do you feel like you got the support that you needed?<br>• What extra support would have helped you to cope with this experience?
Looking at the future, what do you think needs to change, in terms of support services, to better address the support needs of men who've had experiences like yours?	• What needs to change in terms of society?<br>• What do you think the most common misunderstandings are when it comes to men's experiences of IPV?<br>• Do you think the general publics view of IPV is?<br>• What do you think the general public's view of IPV is when it comes to men's experiences?<br>• What needs to change in terms of policy (includes government organisations, PSNI, etc.)
What motivated you to take part in the study? And what was your experience taking part in this study like this for you?	N/A

Finally: Is there anything else that you haven't mentioned that you would like to tell us about?

THANK YOU FOR YOUR TIME
